# A Dual‐Color Fluorescent Reporting System for Dynamic Monitoring of Microglial Phagocytosis of Amyloid β Oligomers

**DOI:** 10.1002/alz.085791

**Published:** 2025-01-03

**Authors:** Xin Huang

**Affiliations:** ^1^ Florey Institute of Neuroscience and Mental Health, Parkville, VIC Australia

## Abstract

**Background:**

The reduced phagocytosis of amyloid β (Aβ) by microglia is linked to increased cognitive decline in Alzheimer’s disease (AD) patients. Previous methods utilized anti‐Aβ antibodies and flow cytometry to reveal Aβ surface binding without internalization. This study introduces a “Two‐Color Fluorescent Reporting System” to overcome limitations, allowing differentiation between intra‐ and extracellular Aβ.

**Method:**

This novel system utilizes AF647‐conjugated Aducanumab to visualize the surface‐bound amyloid β oligomers (Aβo), while pHrodo red serves to delineate intra‐lysosomal Aβo. Initial investigations focused on pHrodo‐Aβo phagocytosis by neuronal (HT22) and glial (U251) cells, comparing them with microglial cells (BV2). Subsequent examinations assessed the impact of glatiramer acetate (GA), a phagocytosis‐promoting peptide, on BV2 phagocytosis of Aβo in comparison to control without GA (basal) and oxidized ATP (oxATP), a P2X receptor antagonist. Flow cytometry was employed for temporal observations at 0.5, 1, 2, 4, and 24 hours, complemented by real‐time fluorescent microscopy for continuous monitoring of the entire process.

**Result:**

In the initial study, BV2 cells exhibited Aβo surface adhesion and subsequent internalization after 2 hours, with the majority phagocytosed into lysosomes by 24 hours. Conversely, HT22 cells displayed surface adhesion of pHrodo‐Aβo without internalization, and after 24 hours, a modest internalization was observed. U251 cells demonstrated weak internalization after 2 hours and more apparent internalization after 24 hours, distinct from BV2. In the second study, the intralysosomal Aβo vs. surface Aβo ratio revealed significant differences between the basal, GA, and oxATP groups after two and 24 hours. Notably, the GA group exhibited increased pHrodo red‐Aβo in a completely acidified state after 24 hours.

**Conclusion:**

This study introduces an innovative dual‐color fluorescent reporter system, showcasing its potential for standardization in elucidating the intricate dynamics of microglial phagocytosis of Aβo. The results underscore the utility of this system, offering promise for advancing research and enhancing our understanding of Alzheimer’s disease pathology.

